# Kettle and Pot

**Published:** 1888-07

**Authors:** 


F. E. DANIEL, M. D., EDITOR AND PUBLISHER.

THIS JOURNAL, by unanimous vote of the members, was made the Official Organ of the Austin District Medical Society.

COLLABORATORS:

*Dr. R. M. Swearingen, Austin.*
*Prof. B. E. Hadra, M. D., Galveston.*
*Prof. Geo. Cupples, M. D., San Antonio.*
*Dr. T. C. Osborn, Cleburne, Texas.*
*Dr E. J. Doering, Chicago.*
*Dr. E. J. Beall, Fort Worth.*
*Dr Odo Betz, Germany.*
*Dr. J. W. McLaughlin, Austin.*
*Dr. T. J. Tyner, Austin.*
*Prof. J. F. Y. Paine, M. D., Galveston.*
*Dr. R. H. L. Bibb, Mexico.*
*Dr. T. J. Bennett, Austin.*
*Dr. Bat Smith, Wharton.*
*Dr. E. Meierhof, New York.*
*Prof. W. B. Rogers, M. D. Memphis, Tenn.*

### NOTICE TO SUBSCRIBERS.

This number begins volume four of the JOURNAL. All of the original subscribers, *i. e.*, those whose subscription begins with number one—July—are notified of the expiration of their paid subscription, and are respectfully invited to renew *as promptly as possible.* We need the money ;—yet knowing that *pay day* in the country comes usually only with the harvest time for the cotton crops, we will cheerfully indulge those who are not prepared to pay at present. There are very many, however, who can find a $2 bill at any season, and to such we say, "we will be much pleased to hear from you; we give you a good journal, and spare no pains or expense; help us pay for it."

### KETTLE AND POT.

The *N. Y. Medical Record* complains that "under the fostering care of the ethics of the American Medical Association, a system of specialty advertising has sprung up amongst the physicians of the West." Under the fostering care of what system of ethics sprung up the *Bulletin-Physician-Advertising-Racket?* and the Interview-advertising-of-cancer-cure-specialty ?

The Journal of the A. M. A. intimates that a distinguished New York Doctor, not remotely connected with the *Record*, is a genius-at-getting-himself interviewed;—and that he had *almost* gotten himself interviewed (on cancer) to the extent of being "sent for" by Emperor Frederick;—at least that there was a hint thrown out in an "interview," that if he *were* "sent for," he could'nt go!—A *modest man* is Shrady!

---

THE TRANSACTIONS T. S. M. A. will be issued next month, from the Steam Job Printing establishment of Eugene von Boeckmann, Austin, the same house whence issued the very creditable volumes of the last year or so.

It is desired to call attention of authors of papers to the actual necessity of getting up their papers in such shape, at least, as to be readable. It is surprising that some authors of really good papers are so slipshod and indifferent as to send their articles in, written in pencil, with words half written, and with such abbreviations as "no" for number; etc. These find an early grave in the waste basket. Others are written on *both sides* of the sheet, a violation of the rules of all printing establishments, which surely condemns the article to the scrap-bag. The Publishing Committee carefully edit the papers which are selected, and go so far as to correct errors—they are very accommodating, but it is not to be expected that they will re-write a paper of twenty or thirty pages this kind of weather. Some papers are beautifully gotten up, and are type-written. It is a luxury to edit such contributions.

---

MARRIED—TYNER-DENISEN—In Memphis, Tenn., Wednesday, July 25, by the Rev. Dr. Daniel: Dr. T. J. Tyner, of Austin, to Miss Emma Denisen, of Memphis. No cards. Dr. Tyner and his accomplished bride left Memphis for New York immediately after the ceremony and will sail for Europe August 1.

Dr. Tyner is the regular delegate of the Texas State Medical Asssociation to the British Medical Society and will attend its meeting in Glasgow, Scotland, September 7. After which the bridal couple will make the tour of Europe and the Continent, the doctor visiting the hospitals of all the great medical centres Berlin, Vienna, Paris, London, Edinburg, etc. From these points he will contribute letters to this JOURNAL, and will return with his bride to Austin late in October. Our sincere congratulations are tendered our distinguished friend, and his bride and they have the JOURNAL'S best wishes for a pleasant journey—both through Europe, and—through life; *Bon voyage.*

MEMBRANOUS CROUP IN A CHILD THIRTEEN MONTHS OF AGE RECOVERY.—Our young friend, S. W. English, M. D., of Hollis, writes us of an interesting case of membranous croup in a child, thirteen months of age, which he treated successfully with alum, steam, warm water, (104° F.), baths, stimulants and milk. The doctor lays great stress on the efficacy of alum in causing the false membrane to be thrown off. He also emphasizes the value of warm baths in extreme dyspnœa and cyanosis. On several occasions in the present case, the Doctor thought tracheotomy would have to be performed, but upon replacing the child in the warm bath relief came every time. He also claims that this is the first case on record of recovery from membranous croup in one so young.

---

THE Secretary of the Texas State Medical Association calls attention of members to the circular letter of President Paine; this will be sent out in circular form immediately after this number of the JOURNAL, and as every *delinquent knows* he is *delinquent*, and just how much he owes, it is hoped those whom the cap fits, will accept it as *intended* for them. If he can do no better let him remit *one year's* dues at least, and this will entitle him to receive a copy of the Transactions. A word to the wise; those who do not help pay for it, should not be disappointed if they do not receive a copy.

Remit to J. Larendon, M. D., Treasurer, Houston, and not to the Secretary.

---

THE BULLETIN RACKET.—It is to be hoped that now "Washington Matthews," "H. C. Yarrow," *et al*, have out-Shradied Shrady *et al.* in the Bulletin-advertising-scheme, and pushed the thing in Sheridan's case to the point of exciting emesis,—a reaction will follow, and the practice will be abolished. The bulletins are intended to let the people know who are considered *the* "leading physicians," it being supposed that distinguished citizens will select as their attendants the most distinguished of the profession. Strange infatuation! A *celebrated* physician needs no *puffing*.

---

WE REGRET to learn that our esteemed friend, Dr. Frank A. Tompkins, of Sandy Point, was cowardly and brutally assaulted recently, and dangerously wounded. He had had some words with a person named Day, but it had been amicably adjusted, apparently,—but while Dr. Tompkins was returning home from a professional visit, the ruffianly fellow *slipped up behind him*, and *shot him in the back* with a load of buckshot! At last accounts the attending physicians thought there was a chance for Dr. Tompkins' recovery—a mere chance. The Doctor is a chivalrous South Carolina gentleman, a ripe scholar, and an experienced physician,—one of the best citizens of his county, and his large circle of friends are grieved to hear of his severe misfortune.

---

DEATH OF DR. PURNELL.—Many of the *Alumni* of the Old University of Louisiana Medical College will be grieved to learn of the death of Dr. H. W. Purnell, familiarly known to a large circle of friends as "Tennie." This most estimable gentleman and physician died at Holly Springs, Mississippi, July 3d inst. He was a most genial companion and friend, and an ornament to the medical profession. He was in his 50th year. "Green grow the grass o'er thy grave," dear Tennie Purnell.

